# Aging Knowledge Moderates How Aging Anxiety Relates to Expectations of Counseling Clients with Alzheimer’s Disease

**DOI:** 10.1093/geroni/igaf122.3261

**Published:** 2025-12-31

**Authors:** Mackenzie Kirby, Grace Caskie

**Affiliations:** Lehigh University, Bethlehem, Pennsylvania, United States; Lehigh University, Bethlehem, Pennsylvania, United States

## Abstract

As the aging population grows, psychologists must be prepared to meet older adults’ psychological needs, including those with neurocognitive disorders such as Alzheimer’s disease (AD). Because few psychology trainees receive any aging-focused training, their clinical expectations for counseling older adult clients with AD may be negatively influenced by a lack of knowledge about aging and by their aging anxiety, which could compromise quality of care. This study examined how trainees’ aging anxiety related to their expectations about counseling an older adult client with AD and whether knowledge about aging moderated this relationship. Doctoral trainees (N = 188; 21-35 years) in clinical and counseling psychology completed measures assessing aging anxiety, knowledge about aging (biological, psychological, social domains), and expectations about counseling an older adult recently diagnosed with AD, described in a case vignette. Regression analyses found that greater aging anxiety (surrounding psychological concerns and fear of old people) predicted trainees’ perceptions of this client as less open to therapy, having less motivation to engage in therapy, and having less responsibility for their therapy. Moderation analyses indicated having greater psychological aging knowledge made trainees more resilient to the negative influence of psychological concerns about aging on expectations of the client, however with low biological aging knowledge, less fear of old people was related to more positive clinical expectations of the client. These findings highlight the importance of integrating targeted geropsychology education into psychology training to reduce the influence of aging anxiety on clinical expectations and improve preparedness for counseling older adults with AD.

